# The Antibacterial Activity of Date Syrup Polyphenols against *S. aureus* and *E. coli*

**DOI:** 10.3389/fmicb.2016.00198

**Published:** 2016-02-26

**Authors:** Hajer Taleb, Sarah E. Maddocks, R. Keith Morris, Ara D. Kanekanian

**Affiliations:** ^1^Department of Healthcare and Food, Cardiff School of Health Sciences, Cardiff Metropolitan UniversityCardiff, UK; ^2^Department of Biomedical Sciences, Cardiff School of Health Sciences, Cardiff Metropolitan UniversityCardiff, UK

**Keywords:** *Phoenix dactylifera* L., date syrup, polyphenol, *S. aureus*

## Abstract

Plant-derived products such as date syrup (DS) have demonstrated antibacterial activity and can inhibit bacteria through numerous different mechanisms, which may be attributed to bioactive compounds including plant-derived phenolic molecules. DS is rich in polyphenols and this study hypothesized that DS polyphenols demonstrate inherent antimicrobial activity, which cause oxidative damage. This investigation revealed that DS has a high content of total polyphenols (605 mg/100 g), and is rich in tannins (357 mg/100 g), flavonoids (40.5 mg/100 g), and flavanols (31.7 mg/100 g) that are known potent antioxidants. Furthermore, DS, and polyphenols extracted from DS, the most abundant bioactive constituent of DS are bacteriostatic to both Gram positive and Gram negative *Escherichia coli* and *Staphylococcus aureus*, respectively. It has further been shown that the extracted polyphenols independently suppress the growth of bacteria at minimum inhibitory concentration (MIC) of 30 and 20 mg/mL for *E. coli* and *S. aureus*, and have observed that DS behaves as a prooxidant by generating hydrogen peroxide that mediates bacterial growth inhibition as a result of oxidative stress. At sub-lethal MIC concentrations DS demonstrated antioxidative activity by reducing hydrogen peroxide, and at lethal concentrations DS demonstrated prooxidant activity that inhibited the growth of *E. coli* and *S. aureus*. The high sugar content naturally present in DS did not significantly contribute to this effect. These findings highlight that DS’s antimicrobial activity is mediated through hydrogen peroxide generation in inducing oxidative stress in bacteria.

## Introduction

*Staphylococcus aureus* is affiliated to chronic wounds that have a strong association with chronic inflammation leading to high morbidity (Orsi et al., 2002). Furthermore, the increase in antibiotic-resistant bacteria poses a threat to health care worldwide resulting in a revived interest in plant products as adjunct antimicrobial agents to control pathogenic microorganisms (Cowan, 1999). Naturally derived compounds such as aloe vera, honey and curcumin (De et al., 2009) are gaining popularity as alternative antimicrobial compounds. A major plant group used for traditional medicinal applications is *Phoenix Dactylifera* L., more commonly known as the date palm. Fruit of the date palm have been used customarily in the treatment of intestinal disturbances (Vyawahare et al., 2009). In Egypt, date palm pollen grains have historically been used to enhance fertility (Al-Qarawi et al., 2003). Bauza (2002) has demonstrated that date palm kernels are included in medicinal skin treatment and nomadic tribes in the Middle East have been known to use traditional date syrup (DS) as an antimicrobial agent for wound healing (Tahraoui et al., 2007).

Date products such as DS are used in the food industry as a sweetening alternative and in the production of beverages and alcohol (Aboubacar et al., 2010). More than often, the perceived health benefits for the consumption and utilization in date-derived medicinal concoctions are attributed to the bioactive and nutritious compounds found in DS and date fruit. DS is a rich source of phenolic compounds which are known potent radical scavengers (Vayalil, 2002), various studies addressing the composition of DS have identified significant antioxidant potential (Guo et al., 2003) which may allude to the scientific basis of date fruit and DS’s traditional medicinal application. Numerous phenolic compounds such as polyphenols and flavonoids are antibacterial as a result of their oxidizing potential (Daglia, 2012), which may offer a rationale for date fruit and DS’s medicinal application as an antimicrobial agent.

Whilst it remains unclear as to precisely how the antioxidant scavenging potential contribute to the bacteriostatic and bactericidal activity of DS. Prooxidants are known to cause physiochemical and structural changes to microorganisms that results in growth retardation (Halliwell, 2008).

The challenge to this notion is the ability to determine by which mode of action DS inhibits microorganisms, and which bioactive compounds contribute to this effect. The topic of antioxidants as powerful scavengers of reactive oxygen species (ROS) has recently gained considerable attention in applied food microbiology, food science and technology and cell immunology. The antioxidant/prooxidant activity of secondary metabolites such as polyphenols can depend on factors such as pH, metal-reducing potential, chelating activity, and solubility (Sakihama et al., 2002). Polyphenols have antioxidant activity (radical scavenging, and metal chelating activity) or prooxidant activity depending on environmental conditions, interaction, structural changes and exposure to microorganisms (Yordi et al., 2012). Polyphenols are able to act as prooxidants in systems that utilize redox active metals such as iron and copper. Binding of the polyphenol complex ligand to Fe^3+^, the complex is able to reduce the iron to Fe^2+^ and is oxidized to a semiquinone, which is capable of reducing further Fe^3+^ oxidizing the semiquinone to a quinone. The reduction of Fe^3+^ generates Fe^2+^ that consequently participates in the Fenton reaction and results in ROS generation.

Bacterial aerobic respiration produces oxygen (O_2_) required in cellular energy production (Macvanin and Hughes, 2010). The incomplete reduction of O_2_ by microorganisms during respiration generates ROS including hydrogen peroxide (H_2_O_2_) and the hydroxyl radical (OH^-^). Bacteria that undergo aerobic respiration defend themselves against the oxidative stress associated with the accumulation of ROS such as exposure to polyphenols through several mechanisms one of which is the production of enzymes catalase and superoxide dismutase which combat ROS. Superoxide dismutase reduces OH^-^ to H_2_O_2,_ and catalase consequently converts H_2_O_2_ to water and O_2_. The detoxification process of ROS is efficient and with intracellular H_2_O_2_ concentration being controlled at a steady state value of 0.2 μM in *Escherichia coli* (Brudzynski et al., 2011). Antioxidants such as polyphenols and flavonoids induce bacterial lysis through increased ROS and H_2_O_2_ production.

Given that DS is known to have various bioactive polyphenols, reported as potential antimicrobial agents, this study aimed to identify the bacteriostatic and bactericidal activity of DS against Gram positive and Gram negative bacteria and to establish whether this activity is influenced by (a) DS phytochemical compounds, namely polyphenols, (b) the susceptibility of bacteria to oxidative stress resulting from hydrogen peroxide generated and mediated by the presence of polyphenols, and (c) osmolarity in regards to sugar content is the not the principal factor contributing to the antibacterial activity.

## Materials and Methods

### Standards, Solvents, and Reagents

The following reagents were obtained from Sigma (Sigma–Aldrich, UK): XAD-2 Resin, Folin-Ciocalteu reagent, Butylated hydroxytuolene (BHT), polyvinylpolypyrrolidone (PVPP), gallic acid, and catechin. Acetone and methanol (HPLC grade), Xylenol orange, aluminum chloride, glucose, fructose, and sucrose (analytical grade) and hydrogen peroxide were obtained from Fisher Scientific (UK) and 2,2′-diphenyl-1-picrylhydrazyl (DPPH) was purchased from Merck (Darmstadt, Germany).

### Date Syrup Preparation for Antibacterial Testing

Date syrup was produced from the date fruit cultivar Khadrawi, belonging to the family *Arecaceae*, genus *Phoenix* and species *dactylifera* during the wet seasons of 2012–2013. The DS was raw and unprocessed; it was stored at 4°C on receipt. The DS was unsterile and not immediately suitable for antibacterial susceptibility testing. Therefore, different sterilization methods were undertaken to determine which method was the most ideal for DS with minimum effect on DS’s constituents. Sterilization of DS using the solvent acetone was determined to be most suitable. A 200 g of DS was mixed thoroughly and soaked in 200 mL acetone (Analytical grade) for 48 h at room temperature. After 48 h the homogenous mixture was filtered through Whatman No. 1 filter paper and the solvent was evaporated under rotary evaporation (Bibby RE-100, Bibby Scientific) at 40°C to ensure all acetone was removed. Crude DS extract was rehydrated in nutrient broth (NB) medium and passed through a 0.22 μm filter (Millex-GV, Millipore, UK) and stored at –80°C for analysis, the final concentration resulted in 50 mg/mL DS.

### Preparation of Artificial DS

High Performance Liquid Chromatography (HPLC) analysis was conducted on the sugars present in DS to determine the percentage of individual sugar constituents. Artificial DS per 100 g was prepared by mixing 4.79 g sucrose (7.6% of total), 29.05 g fructose (46.13% of total), and 29.13 g glucose (46.3% of total) in sterile deionised water and warmed in a water bath at 50°C for 10 min to ensure complete dissolving of sugars.

### Extraction of Flavonoid and Phenolic Fraction of DS on XAD-2 Resin

Date syrup (50 g) was mixed with 250 mL of pH2 HCl water for 24 h; the mixture was filtered through cotton wool to remove un-dissolved solid particles. XAD-2 resin (approximately 47 g) was initially conditioned in 2 M HCl for 1 h, conditioned by soaking in 1:1 methanol and water for pre-swelling overnight. The slurry with the resin was packed into a glass column (50 cm^3^) and the solution removed for an approximate bed volume of 1 × 50 cm^3^ and rinsed with 1 L of deionised water.

The filtered DS solution was passed slowly through the packed resin column, followed by 250 mL of acidified water (pH2), deionised water (300 mL), and phenolic fractions were finally eluted with 300 mL pure methanol. A 50 mL of collected methanol extract was concentrated to dryness under vacuum at 40°C, re-dissolved in water and stored at –80°C for analysis, and the remaining methanol extract was stored at –80°C for and dissolved accordingly for antibacterial analysis.

### Determination of Antioxidant Activity

#### Quantification of Total Phenol Content

The total phenolic content of DS was determined by the Folin-Ciocalteu colorimetric assay based on the procedure previously identified by Al-Farsi et al. (2005). Gallic acid was used as a spectrophotometric standard (0–100 mg/mL) and results were expressed and means ± SD mg of gallic acid equivalents (GAE) per 100 g of DS. Measurements were taken in triplicate.

#### Total Flavonoid Content

Total flavonoid content was measured by the aluminium chloride colorimetric assay described by Zhishen et al. (1999). Absorbance was measured at 510 nm against a blank control. Total flavonoid content was expressed as mg GAE per 100 g DS.

#### Total Flavanol Content

Total flavonol content was adapted from the method described by Jimoh et al. (2010); 200 μl of DS (25 mg/mL) was mixed with 250 μl of 2% AlCl_3_ and 250 μl of 5% sodium acetate solution. Mixtures were sealed and incubated for 2.5 h at room temperature. The absorbance was measured at 440 nm and results were expressed as mg of catechin equivalents per 100 g of DS (mg catechin/100 g DS).

#### Total Tannin Content

The total tannin content was determined by the Folin-Ciocalteu method after the removal of tannins by their adsorption to the insoluble matrix PVPP. This method was based on Hagerman et al. (2000) and Kchaou et al. (2013); 1 mL of DS extract (25 mg/mL) was added to 100 mg of PVPP and incubated for 15 min at 4°C. The mixture was vigorously shaken and centrifuged for another 15 min at 13,000 g, where the supernatant was collected and non-adsorbed phenolics were subjected to the Folin-Ciocalteu assay for total phenolic content. Results were subtracted from total phenolic content and total tannins was expressed as mg GAE / 100 g fresh weight.

#### Total Carotenoid Content

Total carotenoids were extracted according to the method of Talcott and Howard (1999), working under red light and in dark conditions total carotenoids were calculated using the following equation and expressed as mg per 100 g of DS:

$$Total\,\;carotenoids=\left[(OD)(V)10^{6}/\left(A^{1}\%\right)(100)\left(W\right)\right]$$

Where OD = absorbance at 470 nm, V = volume of sample extract, A^1^% = the average extinction coefficient for a 1% mixture of carotenoids at 2500, and W = sample weight in g.

#### Total Anthocyanin Content

Total anthocyanin was determined and calculated according to the pH-differential method as described by Giusti and Wrolstad (2001). Total anthocyanin content was expressed as mg/100 g of DS and calculated according to the following two equations:

(1) The difference in absorbance between the two anthocyanin extracts were calculated by:

$$\Delta A=\left({OD}_{510}\,\;PH\,\;1.0-{OD}_{700}\,\;pH\,\;1.0\right)-\left({OD}_{510}\,\;pH\,\;4.5-{OD}_{700}\,\;pH\,\;4.5\right)$$

(2) The monomeric anthocyanin pigment concentration in the original sample is expressed as cyaniding3-glucoside equivalents and calculated on the basis of the following formula:

[(ΔA)(MW)(DF)(V)(100)/(ε)(L)(W)]

Where MW = molecular weight of cyaniding3-glucoside (449.2 g/mol), DF = dilution factor, V = final volume in mL, 𝜀 = molar extinction coefficient for cyaniding3-glucoside (26,900), L = cell path length of 1 cm and W = sample weight in g.

### Evaluation of Antioxidant Activity

#### DPPH Radical Scavenging Activity

DS’s anti radical scavenging capacity was assessed based on the scavenging activity of the stable free radical DPPH. Briefly, 100 μl of different DS concentrations (5–50 mg/mL) dissolved in deionized water were aliquoted into a 96-well plate (Costar), 50 μl of ultrapure (ELGA) water was added followed by 50 μl of 400 μm of DPPH (in absolute ethanol). The plate was sealed and shaken for 5 minutes and subsequently incubated in the dark for 25 min at room temperature. Absorbance was measured spectrophotometrically at 490 nm against a blank solution. The commercially available antioxidant BHT was used as a positive control (10 mg/mL in ethanol) and the percentage inhibition activity was calculated based on the following equation and expressed as % antioxidant activity:

$$\left[{OD}_{1}-{OD}_{2}/{OD}_{1}100\right]$$

where OD_1_ is absorbance of blank control and OD_2_ is absorbance of sample extract.

### Antibacterial Susceptibility Testing

#### Bacterial Strains

*Escherichia coli* (reference strain NCTC 10418) and *S. aureus* (reference strain NCTC 13142) were used throughout the study. Cultures were grown aerobically in NB (Fluka) for 24 h at 37°C to promote planktonic growth.

#### Minimum Inhibitory Concentration (MIC) and Minimum Bactericidal Concentration (MBC)

Minimum inhibitory concentration (MIC) for DS and extracted DS polyphenol against *E. coli and S. aureus* was determined using a broth-micro dilution method and spectrophotometric assay. MICs were determined in sterile 96 well round bottomed polystyrene microtitre plates (Corning Costar Ltd, New York, NY, USA) in accordance to methods of the Clinical and Laboratory Standards Institute [CLSI] (2012) MIC was determined by serial dilution (5–50 mg/mL in increments of 5 mg/mL). Bacterial inoculum corresponding to 0.5 McFarland standard of pre-culture [16 h at 37° C and equivalent to 10^6^ colony forming units (CFU)] was added to test samples at each concentration. Samples were measured in triplicate. Plates were incubated at 37°C for 24 h and turbidity was measured spectrophotometrically at 650 nm in a plate reader (SPECTROstar Nano, BMG Labtech). The MBC was assessed in accordance to Clinical and Laboratory Standards Institute [CLSI] (2012) standards whereby those wells described for the MIC above, showing no apparent growth were streaked onto nutrient agar (NA) (Fluka). Plates were incubated overnight at 37°C, the plates with the lowest concentration of DS and DS polyphenol sample showing no growth following incubation overnight was recorded as the MBC. Tetracycline was used as an antibiotic control with a stock concentration of 33 μg/ml.

#### Measurement of H_2_O_2_ Concentration

The generation of hydrogen peroxide in NB medium without bacterial cells (cell free medium) after the addition of DS, DS polyphenols, or artificial DS for 1 h at 37°C was measured by the ferrous ion oxidation-xylenol orange (FOX) assay as described by Packer and Sies (2001), and Maeta et al. (2007). DS was prepared fresh in NB medium corresponding to concentrations sub-lethal (15 mg/mL) and lethal (30 mg/mL) to bacteria as identified in MIC studies. DS polyphenols and artificial DS were prepared at concentrations of 30 mg/mL to investigate their independent effect on H_2_O_2_ production. A working FOX reagent was prepared from two separate reagents; reagent 1 consisting of 4.4 mM BHT in methanol and reagent 2 compromised of 1 mM xylenol orange and 2.56 mM ammonium ferrous sulfate in 250 mM H_2_SO_4_, reagents were prepared fresh daily for each assay.

Samples of DS, extracted DS polyphenols or artificial DS (90 μl) were mixed with 10 μl of methanol, vortexed, and left to incubate at room temperature for 30 min, 900 μl of working FOX reagent was added to each sample assayed in triplicate and incubated for another 30 min followed by centrifugation at 15,000 *g* for 10 min. Absorbance was read at 560 nm against a methanol blank containing the necessary amount of sample to correct for background associated with sample. The FOX assay was calibrated using standard H_2_O_2_, diluted from stock (500 μM) and its concentration assessed using molar extinction coefficient of 43 M^-1^ cm^-1^ at 240 nm.

### Analysis of Bacterial Survival

*Escherichia coli* and *S. aureus* were cultured in NB medium at 37°C for 16 h in accordance to Clinical and Laboratory Standards Institute [CLSI] (2012) standards, DS, DS polyphenols, or artificial DS with or without 100 U/mL catalase or H_2_O_2_ (1 mmol/L) were added to NB medium and allowed to equilibrate for 4 h. This was followed by inoculation with bacteria corresponding to 10^6^ CFU/mL (0.5 McFarland). After incubation at 37°C for 4 h with shaking, cells were diluted (10^-1^–10^-8^) and enumerated using the surface drop count method to determine CFU.

### Statistical Analysis

All data were expressed as mean ± SD of independent triplicates unless otherwise stated. One way ANOVA with Tukey’s *post hoc* analysis was used for multiple comparisons within groups of normally distributed data Statistical analysis was performed using GraphPad Prism^®^ Version 6 software and results were significant at *p* < 0.05 and *p* < 0.01.

## Results

The antimicrobial activity of DS might be associated with the presence of antioxidative compounds in DS that possess bioactive behavior. It was hypothesized that the phytochemical compounds present in DS may be involved in redox reactions mediated by the production of H_2_O_2_ that results in bacterial inhibition providing justification for DS’s traditional medicinal application.

### Determination of DS Antioxidant Behavior

The determination of secondary metabolites as antioxidants is outlined in **Table 1**. In comparison to previous literature investigating date fruit and DS (Al-Farsi et al., 2007; Dhaouadi et al., 2010; Abbès et al., 2013) the results are in agreement that DS has sufficient secondary metabolites that are typically associated with bioactive behavior and radical scavenging (Vayalil, 2012).

**Table 1:** Antioxidant determination of date syrup (DS).

	DS (mg/100 g)
Total phenol content	605.1 ± 31.6
Tannins	357.4 ± 18.7
Flavanoids	40.5 ± 28.9
Flavanols	31.7 ± 8.6
Anthocyanins	6.63 ± 1.9
Carotenoids	1.59 ± 0.1

*Results are expressed as means ± SD mg/100g of fresh DS weight.*

**FIGURE 1:**
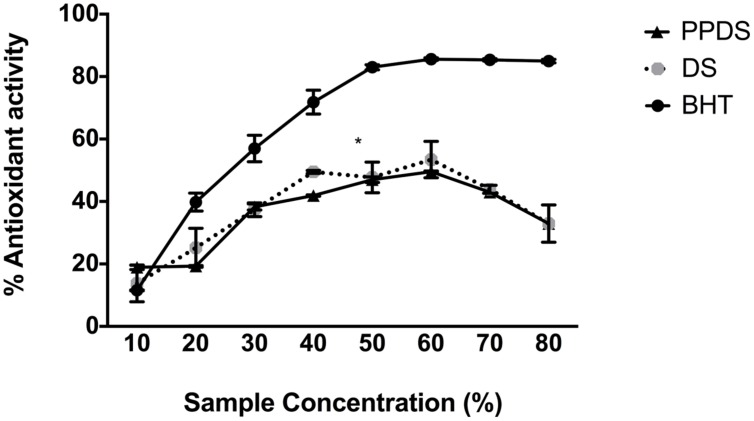
**Comparison of the antioxidant potential of date syrup (DS) (round gray scale dot) and extracted DS polyphenol (PPDS) (black triangle) against the commercially available antioxidant butylated hydroxytuolene (BHT) (black dot).** Results are expressed as mean ± SD of three independent experiments and significant difference between DS treatments and BHT is indicated as ^∗^*p* < 0.05.

As the concentration of DS and DS polyphenols (PPDS) increases, so does the percentage antioxidant behavior, which is a demonstration of the free radical scavenging activity (**Figure 1**). However, this antioxidant behavior was only evident up until a concentration of 60-70% DS, therefore at a greater concentration of DS, the antioxidant power began to decline.

### Antibacterial Susceptibility Testing

Date syrup and extracted DS polyphenols were investigated for their antibacterial activity. The bacteriostatic activity of DS and DS polyphenols was tested against *E. coli* and *S. aureus* and represented as the MIC. The MIC of DS and DS polyphenols is outlined in **Table 2**. DS’s MIC for the tested bacteria was determined at 30 mg/mL. For extracted DS polyphenols, the MIC was 30 mg/mL for *E. coli* and 20 mg/mL for *S. aureus*, these results are not significantly different (*p* < 0.05) from DS’s MIC suggesting that both DS and DS polyphenols exert the same effect in retarding bacterial growth.

**Table 2:** Minimum inhibitory concentration (MIC) and Minimum bactericidal concentration (MBC) of DS, and extracted DS polyphenols necessary to inhibit microbial growth *in vitro* expressed in mg/mL.

Microorganism	DS mg/mL (SD)		DS polyphenols mg/mL (SD)	
	MIC	MBC	MIC	MBC
*Escherichia coli*	30 (±0.83)	40 (±0.97)^∗^	30 (±0.11)	32 (±0.73)^∗^
*Staphylococcus aureus*	30 (±0.76)	35 (±0.54)^∗^	20 (±0.82)	23 (±0.94)^∗^

*Mean and SD of results are expressed as three independent experiments in triplicates. Significant differences between each treatment and microorganism indicated as ^∗^p < 0.05.*

**FIGURE 2:**
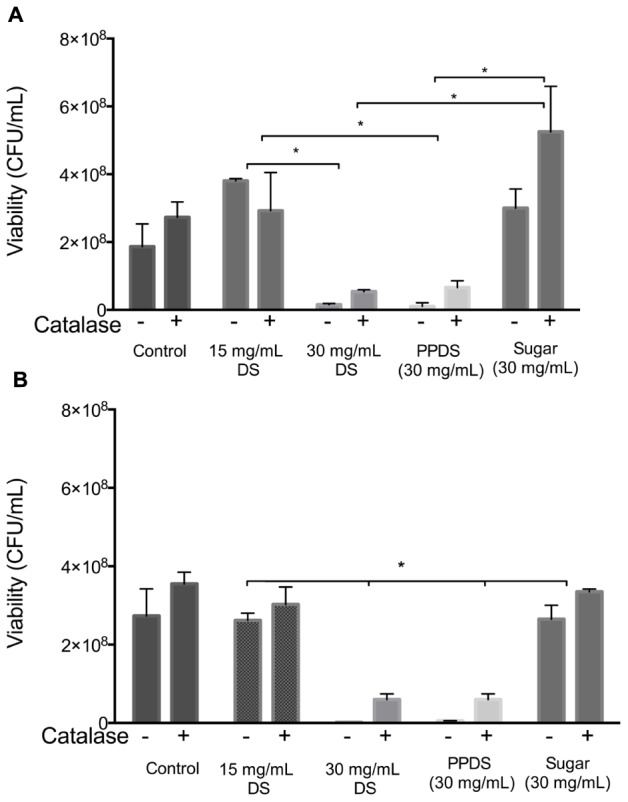
**Inhibitory effects of catalase on the antibacterial action of DS.** DS treatments; DS, extracted DS polyphenols (PPDS) and artificial DS sugar (Sugar) were added to **(A)** *Escherichia coli* and **(B)** *Staphylococcus aureus* cell suspensions with or without 100 U/mL catalase. After being incubated at 37°C for 4 h with shaking, cell viability was determined using surface drop count methods and expressed as viability in colony forming units (CFU). Data is mean ± SD of three independent experiments. Significant differences between treatment groups are indicated as ^∗^*p* < 0.05.

Furthermore, it was found that treatment of both *E. coli* and *S. aureus* with the different DS treatments using concentrations of DS corresponding to sub-MIC (15 mg/mL), above MIC (30 mg/mL), extracted DS polyphenol (PPDS) and a concentration of artificial DS sugar (consisting of 7.6% w/v sucrose, 46.13% w/v fructose, and 46.3% w/v glucose) (Sugar) significantly decreased the survival rates as represented in **Figure 2**.

To assess whether extracted DS polyphenol (PPDS) derived hydrogen peroxide was responsible for the suppression of *E. coli* and *S. aureus* growth, the effect of catalase on the antibacterial activity of the different DS treatments including extracted DS polyphenol and artificial DS sugar was examined. The addition of 100U/mL catalase restored the growth of *E. coli* significantly (*p* < 0.05) and *S. aureus* medium containing different DS treatments as outlined in **Figure 2**. This suggests that H_2_O_2_ mediates the antibacterial activity of DS.

### Hydrogen Peroxide Mediates the Antimicrobial Action of DS

**FIGURE 3:**
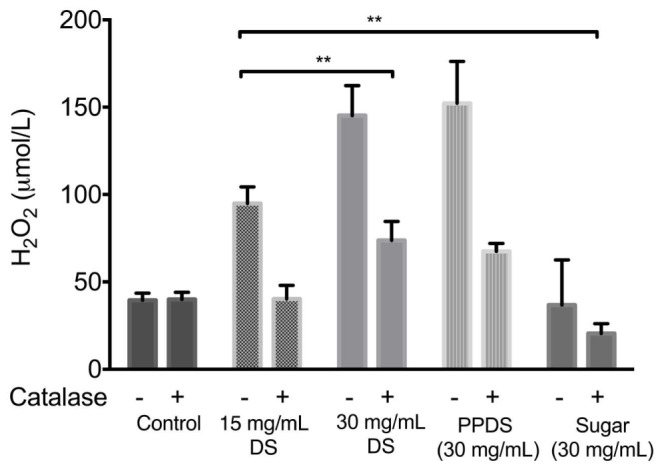
**H_2_O_2_ generation by different sample treatments of DS, extracted DS polyphenols (PPDS), and artificial DS sugar (Sugar) and the effect of 100 U/mL catalase on the production of hydrogen peroxide by DS.** The concentration of H_2_O_2_ in the medium was immediately determined by the FOX method 1 h after the addition of different DS treatments to NB medium (pH7.5). Data is mean ± SD of three independent experiments. Significant differences between treatment groups are indicated as ^∗∗^*p* < 0.01.

To obtain evidence that H_2_O_2_ is generated by DS, the hydrogen peroxide production was determined in NB medium at concentrations of DS corresponding to 15 mg/mL DS, 30 mg/mL DS, extracted DS polyphenol (PPDS) and a concentration of artificial DS sugar (Sugar) corresponding to the MIC, this was achieved by the FOX method an assay sensitive to hydrogen peroxide production by measuring the formation of a complex between xylenol orange and ferric ion as identified in **Figure 3**. The addition of 100 U/mL catalase on the hydrogen peroxide activity of DS was also further investigated, the enzyme catalase quenches the generation of H_2_O_2_ and the addition of catalase significantly (*p* < 0.05) decreased the hydrogen peroxide generated.

The levels of H_2_O_2_ increased significantly as the concentration of DS increased, this was also evident with extracted DS polyphenols. This demonstrated that the addition of catalase had an effect on hydrogen peroxide activity regardless of DS treatment and concentration, and this effect was further corroborated (**Figures 2A,B**) with the addition of catalase in the presence of bacteria. DS sugar appears to generate the least hydrogen peroxide and is influenced least by catalase activity suggesting no direct effect in DS’s antimicrobial activity in inhibiting *E. coli* and *S. aureus*.

**FIGURE 4:**
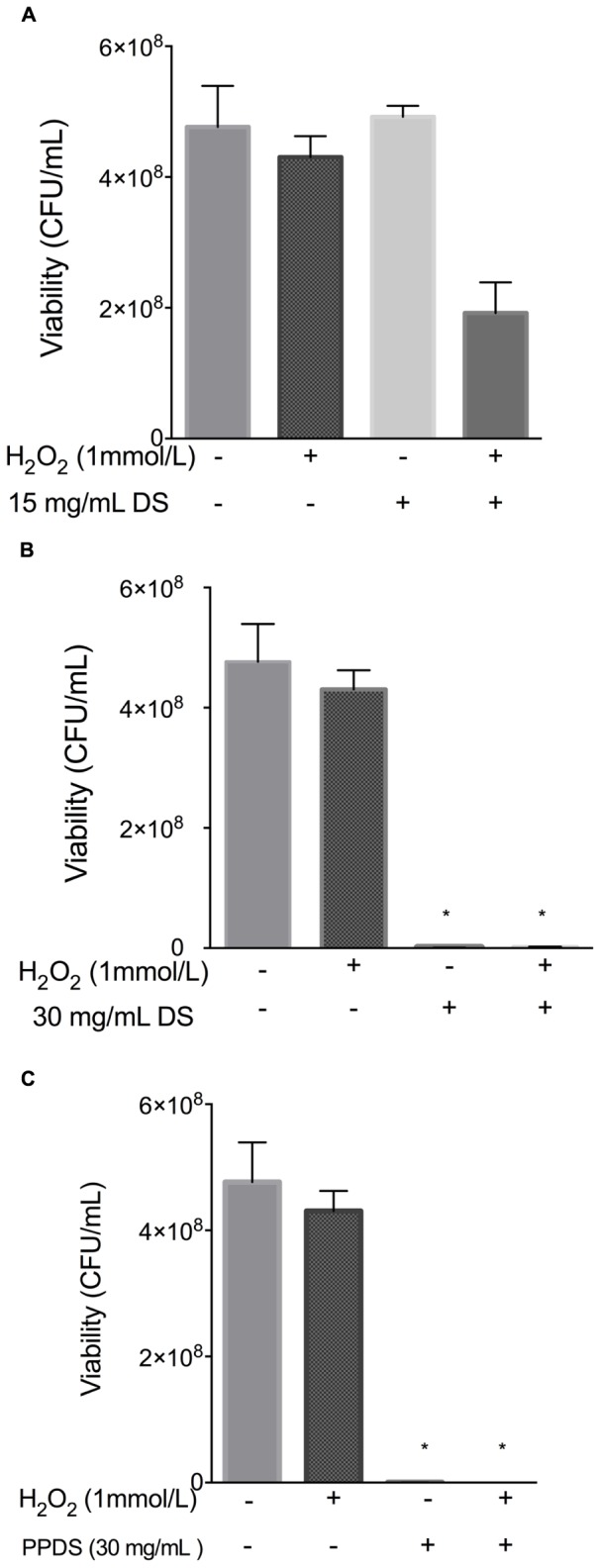
**Evaluation of antioxidant activity of DS treatments. (A)** 15 mg/mL DS, **(B)** 30 mg/mL DS, and **(C)** polyphenol DS (PPDS)(30 mg/mL). DS treatments were added to NB medium and incubated at 37°C with *E. coli* for 4 h with shaking, cell viability was determined using the surface drop count method and expressed as viability in CFU. Significant differences between treatment groups are indicated as ^∗^*p* < 0.05.

When *E. coli* cells were treated with hydrogen peroxide, the addition of 15 mg/mL concentration of DS appeared to behave as an antioxidant, as outlined in **Figure 4**, indicating that this concentration of DS in conjunction with hydrogen peroxide reduced any excessive accumulation of hydrogen peroxide that would otherwise be lethal (**Figure 4A**). Interestingly, a 15 mg/mL concentration of DS appeared to enhance the growth of bacterial cells implying antioxidative behavior, this result was supported by previous MIC’s whereby this concentration is not inhibitory to bacteria and it is possible that the % antioxidant activity as outlined in **Figure 1** is not strong enough to inhibit bacterial growth.

**FIGURE 5:**
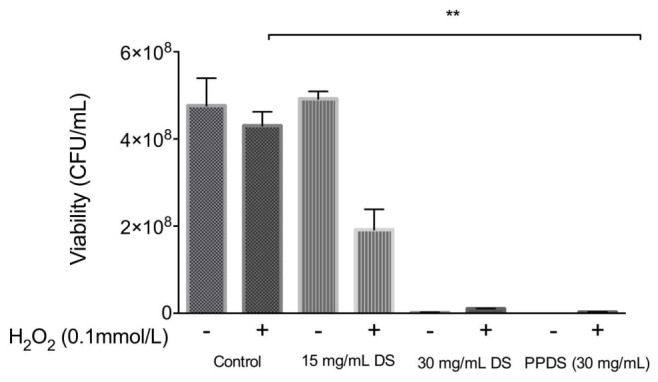
**Evaluation of the synergistic effect of DS and H_2_O_2_ on cellular viability.** Significant differences between treatment groups are indicated as ^∗∗^*p* < 0.01.

Date syrup and DS polyphenol where then further evaluated for synergistic activity with hydrogen peroxide as identified in **Figure 5**.

Date syrup and extracted DS polyphenol at the highest antioxidant activity potential, function as a prooxidants in inhibiting *E. coli*, whereas at a lower concentration it behaves as an antioxidant in allowing bacteria to survive, which corresponds to the MIC values.

## Discussion

This study demonstrated that DS, and DS polyphenols, the most abundant bioactive constituent in DS, have antibacterial activity against the disease causing pathogens *E. coli* and *S. aureus*. The study has also shown that the extracted polyphenols retard bacterial growth and has observed that DS behaves as a prooxidant by generating hydrogen peroxide that mediates bacterial growth inhibition as a result of oxidative stress. Furthermore, low concentrations of DS demonstrated antioxidative activity by reducing hydrogen peroxide, whereas at optimal bacterial growth and weakly alkaline conditions DS demonstrated prooxidant activity that inhibited the growth of *E. coli* and *S. aureus*. The osmolarity as a result of the high sugar content naturally present in DS did not significantly contribute to this effect. These findings highlight that DS and DS polyphenols interaction with bacteria are involved in prooxidant mediated bacterial inhibition.

The determination of secondary metabolites as antioxidants is outlined in **Table 1**. In comparison to previous literature investigating date fruit and DS (Al-Farsi et al., 2007; Dhaouadi et al., 2010; Abbès et al., 2013), the results indicate that DS contains secondary metabolites that are associated with bioactive behavior (Vayalil, 2012).

The extent to which the bacterial growth was inhibited by DS and DS polyphenols was related to the content of redox active phenolic compounds and H_2_O_2_. These results support the assertion that the structural interaction between these bioactive compounds is responsible for growth inhibition beyond an osmotic effect of sugars alone. This offers a new possibility that redox active phenolic compound present in DS; date fruit and other antioxidant rich fruits are active intermediates contributing to microbial impairment.

Although polyphenols and individual phenolic compounds have long demonstrated antioxidant behavior, the present study has demonstrated that DS and DS polyphenols inhibit the growth of *E. coli* and *S. aureus* by generating H_2_O_2_. Furthermore, it has also been demonstrated that DS polyphenols, one of the most abundant constituents in DS, function similarly to whole DS suggesting that DS polyphenols are the major constituents contributing to DS’s antibacterial activity (**Figures 2** and **3**). The capacity for DS and DS polyphenols to generate H_2_O_2_ in culture medium is consistent with current literature (Nakagawa et al., 2004; Yamamoto et al., 2004; Liu et al., 2013) describing H_2_O_2_ generation in various mediums, implying that organic components of medium (such as vitamins, proteins, and inorganic salts) do not directly affect DS and DS polyphenol mediated H_2_O_2_ generation.

Critically, it was also observed that the contribution of osmolarity of DS had no significant influence on its antibacterial activity with MIC for artificial DS being higher than DS or extracted DS polyphenols (**Figures 2A,B**).

Polyphenols are able to inhibit microorganisms and the antimicrobial activity of polyphenols is dependent on their chemical structure and environmental conditions (Almajano et al., 2007). This study investigated whether DS and extracted DS polyphenols function as an antioxidant or as an antimicrobial. The naturally weak acidic DS (pH 5.1) at low concentrations behaved as an antioxidant and protected both *E. coli* and *S. aureus* from H_2_O_2_ induced oxidative damage, whereas at MIC concentrations DS and extracted DS polyphenols demonstrate prooxidant activity (**Figures 4** and **5**) thus behaving as an antimicrobial. The exact mechanism contributing to this effect remains unclear but polyphenols exist as esters of organic acids and can be readily bound to protein (Kroll et al., 2003); the interaction of polyphenols with proteins present in the bacteria result in ionic bonding and hydrogen bonding interactions (Canillac and Mourey, 2004) this will alter protein activity in the microorganism and make it more susceptible to treatment, but will also influence the antioxidant activity of polyphenols (Rawel et al., 2001, 2002).

These observations could be the result of changes to the proteins on and within the bacteria as a result of the interaction with DS and DS polyphenols, making it more susceptible to attack, and oxidative stress. Oxidants such as polyphenols cause oxidative stress and as aerobic bacteria, both *E. coli* and *S. aureus* have evolved intricate molecular mechanisms in response to oxidative stress by the activation of several stress genes (Macvanin and Hughes, 2010; Brudzynski et al., 2012).

Oxidative stress and damage is often associated with DNA damage due to the breakdown of fragments in DNA and further transcriptional changes in antioxidant associated genes such as superoxide dismutase and catalase (Brudzynski et al., 2012), which are induced and influenced by H_2_O_2_. The *oxyR* and *perR* genes control the expression of inducible forms of *katG*, and *ahpCF* genes, which function to homeostatically control the concentration of H_2_O_2_ once it becomes too high. Therefore it can be suggested that the antibacterial activity of DS mediated by hydrogen peroxide will most likely demonstrate transcriptional changes associated with antioxidant genes and oxidative stress genes.

In agreement with previous literature (Brudzynski et al., 2012; Chen et al., 2012; Liu et al., 2013) pre-treatment of DS and extracted DS polyphenols with catalase to remove H_2_O_2_ reduced the bacteriostatic activity of DS to a conservative level (**Figures 2A,B**), this was particularly significant between 15 and 30 mg/mL DS and was independent of the initial H_2_O_2_ concentration (**Figure 3**) thus suggesting that H_2_O_2_ generated as a result of DS induces antibacterial activity.

It has been recently documented that DS is an antioxidant fruit with specific compounds possessing antioxidant activity (Cadenas and Packer, 2005; Dhaouadi et al., 2010), of DS constituents the polyphenol compounds are renowned for their antioxidant behavior, **Figure 1** illustrates this behavior. As shown in **Figure 1** the antioxidant behavior of both DS and extracted DS polyphenols increase linearly as the concentration increases (*p* < 0.05). This assay revealed two particular insights; firstly, there was no significant difference between DS antioxidant activity and DS polyphenol antioxidant activity which suggests that the polyphenols in DS compromise predominantly the bioactive constituents and these bioactive compounds influence H_2_O_2_ in mediating it as an antimicrobial agent. Secondly, the increase in antioxidant behavior (activity) was observed repeatedly up until 60% (corresponding to 30 mg/mL), further supporting the role of DS and DS polyphenols as both antioxidants and prooxidants in antibacterial activity. At a concentration corresponding to 15 mg/mL, which is sub-lethal MIC, DS demonstrates antioxidative behavior signifying that it scavenges any free radicals and reduces H_2_O_2_ generated thus allowing bacterial cells to proliferate and grow. This is evident in the antibacterial results in both the presence and absence of catalase, signifying that this concentration is not lethal to bacteria implying minimal stress responses are activated by bacteria at this concentration. Previous research conducted on DS and date fruit support this finding (Procházková et al., 2011; Abbès et al., 2013; Kchaou et al., 2013; Martín-Sánchez et al., 2014).

Despite apparent antioxidative activity, this was diminished at concentrations of 60%, above 60% it acts as a prooxidant suggesting high concentration of DS and DS polyphenols are required to achieve prooxidant mediated bacterial inhibition. It is possible that the prooxidant activity and subsequent H_2_O_2_ generation are affiliated with the presence of metal ions. The co-incubation of bacteria with DS polyphenols may disrupt bacterial respiration by sequestering metal ions leading to generation of H_2_O_2_. As a traditional medicinal application, this provides a preliminary scientific basis for DS’s medicinal use as an antimicrobial agent and it’s potential for future bacterial infection treatment. This observation is supported by previous literature highlighting the closely related relationship in polyphenols behaving as prooxidants and antioxidants, suggesting that dietary polyphenols exhibit both antioxidative and prooxidative properties under certain conditions such as pH, metal reducing potential, solubility and a natural defense in response to attack (Sakihama et al., 2002; Perron and Brumaghim, 2009; Procházková et al., 2011). This implies that prooxidant environment is beneficial, since, by imposing a mild degree of oxidative stress, the levels of antioxidant defenses and xenobiotic-metabolizing enzymes might be raised, leading to protection through cytotoxicity in inhibiting microorganisms (Halliwell, 2008).

## Conclusion

It has been demonstrated for the first time that DS and DS polyphenols are able to inhibit Gram negative *E. coli* and Gram positive *S. aureus* by generating H_2_O_2_, and that DS polyphenols are active intermediates directly involved in inducing oxidative stress in bacteria as a result of hydrogen peroxide generation. These results confirm the critical relationship between antioxidants and prooxidants of DS polyphenols in bacterial growth and bacterial inhibition. It has also been shown that the high content of naturally occurring sugars in DS do not significantly contribute to its antibacterial activity. These results confirm the critical role of the relationship of antioxidants and prooxidants of DS polyphenols in bacterial inhibition and as an antimicrobial agent.

## Author Contributions

HT, SM, RM, and AK conceived of the experimental ideas and wrote the paper; HT carried out the experiments.

## Conflict of Interest Statement

The authors declare that the research was conducted in the absence of any commercial or financial relationships that could be construed as a potential conflict of interest.
